# Development of Polycistronic Baculovirus Surface Display Vectors to Simultaneously Express Viral Proteins of Porcine Reproductive and Respiratory Syndrome and Analysis of Their Immunogenicity in Swine

**DOI:** 10.3390/vaccines11111666

**Published:** 2023-10-31

**Authors:** Chao-Yu Hsu, Yun Jang, Wei-Ru Huang, Chi-Young Wang, Hsiao-Wei Wen, Pei-Chien Tsai, Cheng-Yao Yang, Muhammad Munir, Hung-Jen Liu

**Affiliations:** 1 Department of Medical Research, Tungs’ Taichung Metroharbor Hospital, Taichung 435, Taiwan; t4361@ms.sltung.com.tw; 2 Ph.D. Program in Translational Medicine, National Chung Hsing University, Taichung 402, Taiwan; 3 Institute of Molecular Biology, National Chung Hsing University, Taichung 402, Taiwan; ruby5978c@hotmail.com (Y.J.); yanzihikki@hotmail.com (W.-R.H.); 4 The iEGG and Animal Biotechnology Center, National Chung Hsing University, Taichung 402, Taiwan; 5 Department of Veterinary Medicine, National Chung Hsing University, Taichung 402, Taiwan; cyoungwang@dragon.nchu.edu.tw; 6 Department of Food Science and Biotechnology, National Chung Hsing University, Taichung 402, Taiwan; hwwen@nchu.edu.tw; 7 Department of Life Sciences, National Chung Hsing University, Taichung 402, Taiwan; ptsai@dragon.nchu.edu.tw; 8 Graduate Institute of Veterinary Pathobiology, National Chung Hsing University, Taichung 402, Taiwan; yangchengyao@nchu.edu.tw; 9 Division of Biomedical and Life Sciences, Faculty of Health and Medicine, Lancaster University, Lancaster LA1 4YW, UK; muhammad.munir@lancaster.ac.uk; 10 Rong Hsing Research Center for Translational Medicine, National Chung Hsing University, Taichung 402, Taiwan

**Keywords:** porcine reproductive and respiratory syndrome virus, baculovirus surface display vectors, neutralizing antibody, IFN-γ, IL-4

## Abstract

To simultaneously express and improve expression levels of multiple viral proteins of a porcine reproductive and respiratory syndrome virus (PRRSV), polycistronic baculovirus surface display vectors were constructed and characterized. We engineered polycistronic baculovirus surface display vectors, namely, pBacDual Display EGFP(BacDD)-2GP2-2GP4 and pBacDD-4GP5N34A/N51A (mtGP5), which simultaneously express and display the ectodomain of His-tagged GP2-gp64TM-CTD, His-tagged GP4-gp64TM-CTD, and His-tagged mtGP5-gp64TM-CTD fusion proteins of PRRSV on cell membrane of Sf-9 cells. Specific pathogen-free (SPF) pigs were administered intramuscularly in 2 doses at 21 and 35 days of age with genetic recombinant baculoviruses-infected cells. Our results revealed a high level of ELISA-specific antibodies, neutralizing antibodies, IL-4, and IFN-γ in SPF pigs immunized with the developed PRRSV subunit vaccine. To further assess the co-expression efficiency of different gene combinations, pBacDD-GP2-GP3-2GP4 and pBacDD-2mtGP5-2M constructs were designed for the co-expression of the ectodomain of His-tagged GP2-gp64TM-CTD, His-tagged GP3-gp64TM-CTD, and His-tagged GP4-gp64TM-CTD proteins as well as the ectodomain of His-tagged mtGP5-gp64TM-CTD and His-tagged M-gp64TM-CTD fusion proteins of PRRSV. To develop an ELISA assay for detecting antibodies against PRRSV proteins, the sequences encoding the ectodomain of the GP2, GP3, GP4, mtGP5, and M of PRRSV were amplified and subcloned into the pET32a vector and expressed in *E. coli.* In this work, the optimum conditions for expressing PRRSV proteins were evaluated, and the results suggested that 4 × 10^5^ of Sf-9 cells supplemented with 7% fetal bovine serum and infected with the recombinant baculoviruses at an MOI of 20 for three days showed a higher expression levels of the protein. Taken together, the polycistronic baculovirus surface display system is a useful tool to increase expression levels of viral proteins and to simultaneously express multiple viral proteins of PRRSV for the preparation of subunit vaccines.

## 1. Introduction

The porcine reproductive and respiratory syndrome virus (PRRSV) belongs to the *Betaarterivirus* genus of the family Arteriviridae and is an enveloped, single-stranded, positive sense RNA virus with a genome of approximately 15Kb in length [1]. PRRSV contains at least 10 open reading frames (ORF) [2,3,4,5,6], of which ORFs 1a and 1b constitute approximately 75% of the viral genome and are translated to produce two large polyproteins (i.e., pp1a and pp1ab) [7,8,9], and ORFs 2–7 can translate into viral structural proteins [10,11]. The pp1a and pp1ab polyproteins are processed by viral proteases to release 14 non-structural proteins, which include four proteases (NSP1α, NSP1β, NSP2, and NSP4), the RNA-dependent RNA polymerase (NSP9), a helicase (NSP10), and an endonuclease (NSP11) [7,8,9]. ORFs 2–5 encode glycosylated membrane proteins GP2-GP5, ORF6 encodes a non-glycosylated membrane protein (M), and ORF7 encodes the nucleocapsid (N) protein [10,11]. ORF2b is enclosed fully within ORF2 and encodes the small, non-glycosylated E or 2b protein [4].

The PRRSV was emerged simultaneously in Europe and North America in the early 1990s and has since become a problem to the swine industry worldwide [12,13,14,15]. PRRSV is divided into two genotypes, *Betaarterivirus suid 1* (formerly known as PRRSV-1 or PRRSV-EU) and *Betaarterivirus suid 2* (formerly known as PRRSV-2 or PRRSV-NA) [16,17]. These two genotypes share only approximately 60% nucleotide sequence identity [12,13]. PRRSV causes a persistent and severe diseases that are characterized by severe reproductive failure in sows and respiratory disease in young pigs as well as weight loss and poor growth [18,19,20,21,22]. Additional clinical signs include fever, blue ear, weight loss, poor growth, diarrhea, stillbirth, dyspnea, and pneumonia. In late pregnancy, sows show clinical symptoms such as abortion, stillbirth, and mummified fetuses [18,19,20,21]. The mortality rate of infected piglets is extremely high, causing serious economic losses to the global pig industry [18,19]. The PRRSV was diagnosed in Illinois, USA in 1987 [23], and the invasion of PRRSV was confirmed in Taiwan in 1993 [24]. The current PRRSV strains in the fields in Taiwan belong to *Betaarterivirus suid 2* (the North American type) [25].

The baculovirus expression system was initially established by Smith and Summer in 1983 [26] and since then has successfully been used for the production of vaccines. The baculovirus gene has a strong promoter, which can produce a large number of exogenous proteins and can carry out protein processing and modification, such as glycosylation and phosphorylation to facilitate optimal protein folding. Thus, the baculovirus expression system is used to produce the recombinant protein that is closer to the original protein in antigenicity, immunity, and biological activity. Importantly, baculovirus does not infect humans and is safe to handle and administer. The system has been proposed to be used as vaccine vectors, RNA interference mediators, gene delivery vectors, and gene therapy [27,28,29,30,31]. The high lethality and widespread distribution of porcine reproductive and respiratory syndrome indicate the need for and importance of PRRSV vaccination. It has previously been demonstrated that structural proteins of PRRSV, including GP2, GP3, GP4, GP5, and M proteins, are critical for virus infection and cell entry and are responsible for the induction of neutralizing antibodies [32,33,34,35,36,37,38] Since several reports suggested that the GP2, GP4, and GP5 proteins of PRRSV are important antigens for inducing neutralizing antibodies [33,34,35,36,37,38] and that the PRRSV GP5 protein mainly induces the production of neutralizing antibodies and the ability of this protein to induce the neutralizing antibody is superior compared to other viral proteins [39]. The aim of this study was to construct baculovirus surface display vectors with multiple expression cassettes to simultaneously express multiple viral proteins and to increase expression levels of viral proteins. To achieve this, two constructs (pBacDD-2GP2-2GP4 and pBacDD-4mtGP5) were designed to display the ectodomain of His-tagged GP2-gp64TM-CTD, His-tagged GP4-gp64TM-CTD, and His-tagged mtGP5-gp64TM-CTD fusion proteins on cell membrane of Sf-9 cells for preparation of subunit vaccines against PRRSV. Our findings revealed that the developed subunit vaccine elicited high levels of ELISA-specific antibodies, neutralizing antibodies, IL-4, and IFN-γ in SPF pigs. Additionally, to further assess the co-expression efficiency of multiple viral genes by the baculovirus surface display system; therefore, the second set of constructs (pBacDD-GP2-GP3-2GP4 and pBacDD-2mtGP5-2M) were designed. Our results revealed that the developed polycistronic baculovirus surface display system is a useful platform to simultaneously express multiple viral proteins and to increase expression levels of proteins. Additionally, this study tested the optimum conditions for expressing PRRSV proteins using the novel baculovirus surface display vectors to further improve the economical production of the PRRSV vaccine.

## 2. Materials and Methods

### 2.1. Cells and Viruses

The CY2-1604 strain of PRRSV (a Taiwanese field isolate; the North American type) was used in this study, which was kindly provided by Professor Chung, National Pingtung University of Science and Technology [40]. The monkey kidney cell line MARC-145 (American type collection ATCC no.CRL-12231) was cultured in Dulbecco’s Modified Eagle’s Medium (DMEM) supplemented with 10% fetal bovine serum (FBS), 1% Penicillin/Streptomycin and 10 mM 4-(2-hydroxyethyl) piperazine-1-ethanesulphonic acid (HEPES) (pH 7. 2) at 37 °C in a 5% CO_2_ humidified incubator. Cells were seeded one day before each experiment in 10-cm culture dishes and cultured until achieving approximately 75% confluence. Cells were infected with PRRSV at 37 °C in a 5% CO_2_ humidified incubator for 3 days. In this work, Spodoptera frugiperda (named hereafter as Sf-9) cells were grown as monolayers in TNM-FH medium (Sigma, St. Louis, MO, USA) supplemented with 10% heat-inactivated fetal bovine serum (FBS; Gibco-BRL, Gaithersburg, MD, USA), 100 U/mL of Penicillin and 100 μg/mL of Streptomycin. Recombinant viruses were propagated and titrated in Sf-9 cells.

### 2.2. Reverse Transcription (RT) and Polymerase Chain Reaction (PCR) and cDNA Cloning

In this work, the signal peptide (SP), transmembrane domain (TM), and cytoplasmic domain (CTD) sequences of GP2, GP3, GP4, GP5, and M genes of PRRSV were removed. Thus, the sequences encoding the ectodomain of GP2 (aa 41–188), GP3 (aa 56–162), GP4 (22-140), GP5 (aa 30–66), and M (aa 1–16) of the PRRSV CY2-1604 strain (GenBank no: MH651737) were amplified by RT-PCR. To carry out RT-PCR, total RNA was isolated from PRRSV-infected cells using TRIzol and RNeasy Mini Kit (Qiagen, Venlo, Netherlands) according to the manufacturer’s protocols. Total RNAs were subjected to RT-PCR assay at 42 °C for 1h using the respective forward primers (20 μM). After inactivation of reverse transcriptase at 95 °C for 2 min, the cDNA was subjected to 35 cycles of denaturation at 94 °C for 1 min, annealing at 58 °C for 1 min, and extension at 72 °C for 1 min and a final extension of 72 °C for 7 min. PCR reaction was carried out in 50 μL reaction containing 1 μL of PRRSV cDNA, 2 μL 2.5 mM dNTP, 2 μL of 10 μM forward primers, 2 μL of 10 μM reverse primers, 5 μL 10× pfu buffer, and 1 μL pfu DNA polymerase. The primers for amplification of the PRRSV genes are shown in Supplementary Table S1. The primer sequences were chosen according to genome sequences of the CY2-1604 strain with required modifications. PCR products were purified and the products were digested with the respective restriction enzymes. To create the respective constructs, purified PCR products containing the ectodomain sequences of the GP2, GP3, GP4, GP5, and M genes were inserted into the corresponding restriction sites of pBacSC, pGEMdual Display, pBacDual Display-EGFP-vector-1, and pET32a vectors, respectively, as described previously [30,41,42]. All plasmids were transformed individually into *E. coli* strain DH5α and the white colonies were selected from Luria-Bertani (LB) agar plates containing ampicillin. The alkaline lysis method was used for mini-preparations of plasmid DNA. All plasmids were checked by both restriction enzyme digestions and DNA sequencing.

### 2.3. Construction of Baculovirus Surface Display Vectors Carrying Multiple Expression Cassettes for Simultaneous Expression of the Truncated Proteins of PRRSV

The detailed procedures for constructing baculovirus surface display vectors with multiple expression cassettes were described previously [31,41,42,43]. Briefly, pBacCE plasmid was created using pFast-Bac DUAL [27]. Sequences encoding the gp64 SS, His6, and multiple cloning sites (XhoI, XbaI, PstI, and EcoRI) located between His6 and baculovirus gp64 TM-CTD were inserted into pBacCE and the resultant plasmid was named pBacSC [30]. To allow rapid identification of the recombinant baculoviruses in Sf-9 insect cells and to eliminate cumbersome and time-consuming assays, the enhanced green fluorescent protein (EGFP) coding sequences were subcloned into the corresponding site in plasmid BacSC under the strong viral polyhedron (polh) promoter [30,31,41,42,43]. EGFP and PRRSV proteins were expressed through different promoters in the same vector. In the present study, the amplified products of GP2 (aa 41–188), GP3 (aa 56–162), GP4 (22-140), GP5 (aa 30–66), and M (aa 1–16) of PRRSV were subcloned into the pBacSC, pGEMdual Display, and pBacDual Display-EGFP-vector-1 vectors, respectively, as described previously [30,41,42]. Construction of the first set of plasmids (pBacSC-mtGP5 and pBacDD-2GP2-2GP4, and pBacDD-4mtGP5) and second set of constructs (pBacDD-GP2-GP3-2GP4 and pBacDD-2mtGP5-2M) were designed. These ectodomains of the His-tagged GP2 (aa 41–188)-gp64TM-CTD, His-tagged GP3 (aa 56–162)-gp64TM-CTD, His-tagged GP4 (aa 22–140)-gp64TM-CTD, His-tagged GP5 (aa 30–66)-gp64TM-CTD, and His-tagged M (aa 1–16)-gp64TM-CTD fusion proteins will be displayed on cell membrane of Sf-9 cells. A previous study has confirmed that the ability of GP5 protein of PRRSV to induce neutralizing antibody is superior to other viral proteins [39]. It is a highly glycosylated protein, and the glycosylation structure causes the epitope to be shielded, which seriously affects the ability to induce neutralizing antibodies. An earlier report suggested that the loss of N-linked glycosylation sites (N34 and N51) in the ectodomain of GP5 enhances both the sensitivity of the virus to in vitro neutralization and the immunogenicity of the nearby neutralization epitope [44]. This finding has great significance for development of PRRSV vaccines of enhanced protective efficacy. Therefore, the amino acid residues N34/51 located in GP5 were substituted with alanine using a Quik Change II site-directed mutagenesis kit (Agilent Technologies Inc., Santa Clara, CA, USA) according to the manufacturer’s instructions. The GP5N34A/N51A mutant (mtGP5) was amplified by RT-PCR and purified PCR products were digested with the respective restriction enzymes followed by subcloning into the pBacSC, pGEMdual Display, and pBacDual Display-EGFP-vector-1 plasmids to create pBacSC-mtGP5 and pBacDD-4mtGP5 constructs, respectively, as described previously [41].

### 2.4. Preparation of Recombinant Bacmid DNA and Construction of Recombinant Baculoviruses

The detailed procedures for preparation of recombinant Bacmid DNA and construction of recombinant Baculoviruses were described previously [41,42,43]. The developed constructs pBacSC-mtGP5, pBacDD-2GP2-2GP4, pBacDD-4mtGP5, pBacDD-GP2-GP3-2GP4, and pBacDD-2mtGP5-2M were verified by enzyme digestion and DNA sequencing. To create genetic recombinant baculoviruses (BacSC-mtGP5, BacDD-2GP2-2GP4, BacDD-4mtGP5, BacDD-GP2-GP3-2GP4, and BacDD-2mtGP5-2M), the developed pBacSC-mtGP5, pBacDD-2GP2-2GP4, pBacDD-4mtGP5, pBacDD-GP2-GP3-2GP4, pBacDD-2mtGP5-2M, and the non-recombinant plasmid pBacCE were transformed into DH10 Bac^TM^ E. coli. Homologous recombination was performed using the helper vector, and the successful recombinant bacmids would destroy the lac Z gene (Bac-to-Bac baculovirus expression system; Thermo Fisher Scientific Co, Taichung, Taiwan). The medium containing X-gal was used for screening blue and white colonies. After two rounds of blue/white selection, recombinant bacmids were isolated from white colonies according to the manufacturer’s instructions (Invitrogen, Carlsbad, CA, USA). The recombinant clones were checked for the presence of the insert by PCR. Positive colonies were cultured in order to isolate the bacmid DNA. The recombined baculoviruses bacmid mtGP5, GP2-2GP4, 4mtGP52, GP2-GP3-2GP4, and mtGP5-2M were transfected into Sf-9 insect cells to construct the genetic recombinant baculoviruses BacSC-mtGP5, BacDD-2GP2-2GP4, BacDD-4GP5, BacDD-GP2-GP3-2GP4, and BacDD-2mtGP5-2M, respectively. The Sf-9 cells were cultured at 27 °C in Sf-900 II SFM. A total of 9 × 10^5^ cells were seeded in 35-mm wells of a six-well plate and allowed to attach for 1 h before transfection. Transfected Sf-9 cells were incubated for 5 h at 27 °C and replaced with fresh medium. After incubation for 48 h at 27 °C, recombinant viruses were selected based on GFP expression and purified by three rounds of plaque isolations. Individual recombinant viruses were titrated by plaque assay and high titer stocks were used for infection of the cells.

### 2.5. Confocal Microscopy

Confocal microscopy was performed as described previously [30,41]. Sf-9 cells were cultured on sterile cover slips (placed in six-well plates) and infected at an MOI of 10. Two days post infection, the cells were fixed by methanol/acetone (v:v = 1:1) for 5 min at −20 °C, rinsed with PBS, and blocked with 2% bovine serum albumin for 30 min at 37 °C. The cells were then incubated with the primary antibody (anti-mouse IgG, 1:3000 dilution) for 1 h at 37 °C, followed by three PBS washes. The cells were subsequently incubated with the secondary antibody (FITC-conjugated goat anti-mouse IgG, 1:50 dilution) for 1 h at 37 °C, followed by three PBS washes. Negative control cells were treated the same way. Protein localization was visualized using a confocal microscope (LSM 510 META, Zeiss, Jena, Germany).

### 2.6. Optimum Conditions for Production of Viral Proteins

To study the optimum conditions for culturing recombinant baculoviruses-infected Sf-9 cells, different culture conditions were assessed. In the present study, various MOIs (10, 20, 30, 40, and 50 MOIs), infection times (2 and 3 days), and different percentages (6–10%) of heat-inactivated FBS were tested.

### 2.7. Expression of PRRSV Proteins in E. coli and Establishment of Enzyme-Linked Immunosorbent Assay (ELISA)

To establish the PRRSV ELISA, the ectodomain of TrxA-His-GP2 (aa 41–188), TrxA-His-GP4 (aa 22–140), and TrxA-His-GP5 (aa 30–66) fusion proteins were expressed in *E. coli* strain BL21 (DE3). The expressed fusion proteins were used as the ELISA coating antigens. The amplified PCR products of the sequences encoding the ectodomain of GP2, GP4, and mtGP5 genes of PRRSV were subcloned into the responding sites of the pET32a expressing vector, respectively. The procedures for PRRSV protein expression in *E.coli* have been described previously [45,46]. Briefly, after induction for 3 h with isopropyl β-d-1-thiogalactopyranoside (IPTG) at a final concentration of 0.4 mM in culture medium, *E. coli* BL21(DE3) containing the constructs were induced, and the final pellets were separated in a 12% SDS-polyacrylamide (SDS-PAGE) gel. For the His-tagged fusion proteins, cells were harvested by centrifugation, followed by resuspension in pET system lysis buffer (20 mM Tris-HCl, pH 8.0, 300 mM NaCl, 0.2 mM PMSF, 10% glycerol, 5 mM imidazole) and sonicated. The cell suspension was centrifuged at 12,000× *g* for 20 min at 4 °C. After centrifugation, soluble proteins were collected for use in the ELISA. To obtain more soluble forms of His-tagged fusion proteins, the transformed *E. coli* BL21 (DE3) were harvested by centrifugation, followed by resuspension in lysis buffer (1PBS, 0.2mM PMSF, 1% Triton X-100, 0.5% sodium lauroyl sarcosinate) [42,43,44,45]. After sonication, the cell suspension was centrifuged at 12,000× *g* for 20 min at 4 °C. Cells were collected by centrifugation at 4500 rpm for 10 min and the supernatant was removed, and the pellet was resuspended in 10 mL of lysis buffer (20 mM Tris-HCl, 0.5 M NaCl, 5 mM imidazole) and sonicated. The cell suspension was centrifuged at 13,000 rpm for 20 min at 4 °C, and soluble proteins in the supernatant were collected and stored at −80 °C for later use. To purify the expressed proteins, the collected supernatant was applied to a nickel column. After washing beads with 150 mL of washing buffer, the His-tagged fusion proteins were eluted from the affinity column with elution buffer (20 mM Tris-HCl, pH 8.0, 300 mM NaCl, 0.2 mM PMSF, 10% glycerol, 200 mM imidazole).

For insoluble proteins, the pellets were resuspended in 10 mL of lysis buffer (20 mM Tris-HCl, 0.5 M NaCl, 5 mM imidazole, 6 M urea), and filtered through the 0.45 µm filter. The supernatant was applied to a nickel column. After washing beads with 150 mL washing buffer, the His-tagged fusion protein was eluted from the affinity column with elution buffer (20 mM Tris-HCl, pH 8.0, 300 mM NaCl, 0.2 mM PMSF, 10% glycerol, 200 mM imidazole). Finally, the purified fusion proteins were dissolved in 50 mM Tris-HCl (pH 8.0) buffer and stored at −80 °C for later use. The protein concentration was determined according to the Lowry method [47]. To determine the optimum dilution of PRRSV antigens in ELISA, checkerboard titrations were carried out as described previously [46]. Purified viral proteins were used at a two-fold serial dilution (0.25–16 μg) in 0.05 M carbonate buffer (pH 9.6) and 50 μL of antigen was added to each well of the ELISA plate. Antigen was coated onto the wells by incubation at 4 °C overnight. Standardization of the ELISA procedures was described previously [46].

### 2.8. SDS-PAGE and Western Blot Assays

The infected cell lysates were subjected to 12% SDS-PAGE and then transferred to PVDF membrane (GE Healthcare Life Sciences, Chicago, IL, USA). Protein expression levels were examined using the appropriate primary antibody and horseradish peroxidase-conjugated secondary antibody. The results were detected on X-ray films (Kodak, Rochester, MN, USA) after membrane incubation with the enhanced chemiluminescence (ECL plus) reagent (Amersham Biosciences, Little Chalfont, England). The quantity of target proteins was calculated using ImageJ.

### 2.9. Immunization of SPF Pigs and Analysis of the Levels of Serum and Neutralizing Antibodies

Sf-9 cells were infected with the genetic recombinant baculoviruses at an MOI of 20 and harvested 3 days post infection. After centrifugation at 15,000 rpm for 10 min, the genetic recombinant baculoviruses-infected cells, which contain the ectodomain of GP2, GP4, and mtGP5 fusion proteins of PRRSV were collected for preparation of subunit vaccines. Without further purification steps, this could save time and cost of vaccine preparation. In this study, BacDD-2GP2-2GP4- and BacDD-4mtGP5-infected cells (5 × 10^7^ cells) were used as one dose to immunize SPF pigs. In this work, ISA201 (Montanide) is a water-in-oil-in-water adjuvant, which was used as an adjuvant in the pig vaccine administration. Its outer water layer antigens can be easily recognized by the immune system, and the inner water layer antigens can be granulated and slowly released to continuously induce immune responses. The 5-week-old SPF pigs were divided into three vaccine groups, BacDD-GP2-GP4- and BacDD-4mtGP5-infected cells, commercial MLV vaccine (Ingelvac PRRSV-2 MLV, Boehringer Ingelheim, Ingelheim, Germany), and negative control group (BacCE). Each immunization group containing three SPF pigs were immunized at the base of the ear by the intramuscular route with the above immunogens, respectively. In this work, PRRSV proteins-specific ELISA antibody titers, serum neutralization (SN) titers, and the levels of IL-4 and IFN-γ cytokines were analyzed. Serum samples were collected every week after the first immunization to determine the PRRSV proteins-specific ELISA antibody titers. SN titers were analyzed at 11 weeks post vaccination. The levels of IL-4 and IFN-γ cytokines were analyzed using IL-4 and IFN-γ ELISA kits. The value of OD_405_ nm was measured using ELISA reader.

### 2.10. SN Test and Analysis of IL-4 and IFN-γ Levels in Swine Immunized with Different Antigens

Previous studies have revealed that IFN-γ and IL-4 are important factors in the vaccine-induced cellular immune response [48,49,50]. Therefore, IFN-γ was used as an indicator of cellular immunity. Sera samples were collected at 4 and 11 weeks after primary immunization for detection of cytokine levels and virus neutralization tests. Analysis of IFN-γ and IL-4 by ELISA was performed using IFN-γ and IL-4 ELISA kits (Uscn, Houston, TX, USA). The serum source of this experiment is the 11th week serum of the pig immunization experiment. Firstly, MARC-145 cells were cultured in a 96-well plate, and 10^4^ cells were cultured in each well. The 2-fold serial dilutions were performed, followed by the addition of 70 μL of 200 TCID_50_ PRRSV HF6-2 strain (a Taiwanese field isolate; the North American type), which was kindly provided by Professor Chung, National Pingtung University of Science and Technology [51]. into each well and incubation at 37 °C for 1 h. The culture medium in the 96-well plate was dry, and 100 μL of PBS was added to each well to wash the cells. The serum and PRRSV virus were mixed evenly and added to a 96-well plate, cultured for 4–6 days, and the CPE was observed. Finally, the neutralizing antibody titer was calculated by the Reed-Muench method [52]. Sera samples were also collected every week after primary immunization for detection of the PRRSV proteins-specific ELISA titters. All pigs were confirmed without antibodies against PRRSV before used.

### 2.11. Ethics Statement

The animal experimental protocols used in this study were approved by the Research Ethics Committee of National Chung Hsing University. All animal experimental procedures were carried out according to the Regulations for the Administration of Affairs Concerning Experimental Animals approved by Council of Agriculture, Taiwan.

### 2.12. Statistical Analysis

All data obtained in this study were analyzed using an independent sample *t* test and expressed as averages of three independent experiment.

## 3. Results

### 3.1. Construction of Baculovirus Surface Display Vectors for the Expression of the Ectodomain of GP2, GP4, and mtGP5 Genes of PRRSV

**Figure 1:**
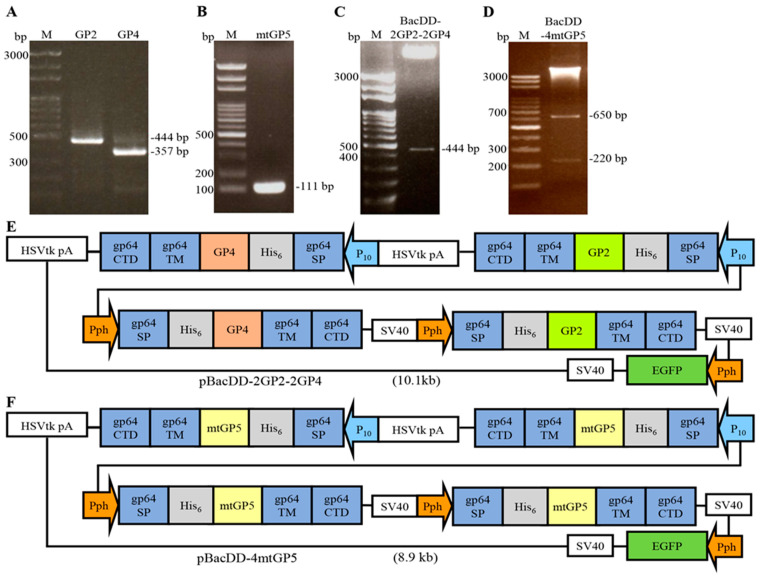
PCR amplification of the sequences encoding the ectodomain of GP2, GP4, and mtGP5 of PRRSV and schematic illustration of novel baculovirus surface display vectors. (**A**,**B**) DNA electrophoresis analysis of PCR products of the truncated GP2, GP4, and mtGP5 genes of PRSSV. Lane M represents DNA marker (Bio 100 bp DNA ladder). (**C**,**D**) pBacDD-2GP2-2GP4 and pBacDD-4mtGP5 constructs were digested with SalI/BamHI and XhoI/KpnI, respectively, to confirm the correctness of the recombinant fragments. (**E**,**F**) Schematic illustration of baculovirus surface display vectors carrying multiple expression cassettes. The gp64 TM-CTD and the gp64 signal peptide sequences of baculovirus localized in the vectors are shown. The developed baculovirus surface display vectors pBacDD-2GP2-2GP4 (**E**) and pBacDD-4mtGP5 (**F**), which carry multiple expression cassettes were shown.

The coding sequences (ectodomain) of GP2, GP4, and mtGP5 genes of PRRSV were amplified by RT-PCR. The PCR products were analyzed by electrophoresis in a 1.2% agarose gel and the expected sizes of the PCR products were seen (Figure 1A,B). The amplified PCR products of the ectodomain of mtGP5 gene of PRRSVwere digested with the respective restriction enzymes, followed by subcloning into the pBacSC, pGEMdual Display, pBacDual Display-EGFP vector-1. The ectodomain of the GP2, GP4, and mtGP5 genes of PRRSV was inserted between the sequences of gp64 signal peptide and gp64 TM-CTD of these vectors under the control of the p10 or Pph promoter. The plasmid vectors were confirmed by restriction enzyme digestion and DNA sequencing. The resultant pBacDD-2GP2-2GP4 plasmid was digested with SalI and BamHI enzymes, respectively, to confirm the correctness of the recombinant fragment. After electrophoresis analysis in a 1.2% agarose gel, the expected size of the respective target gene fragments was 444 bp (Figure 1C). The constructed pBacDD-4mtGP5 vector was digested with XhoI and KpnI enzymes. After electrophoresis analysis in a 1.2% agarose gel, the expected size of the respective target gene fragments was 220 bp and 650 bp (Figure 1D). Schematic illustration of the developed baculovirus surface display vectors are shown in Figure 1E,F.

### 3.2. Preparation of Genetic Recombinant Baculoviruses BacSC-mtGP5, BacDD-2GP2-2GP4, and BacDD-4GP5

**Figure 2:**
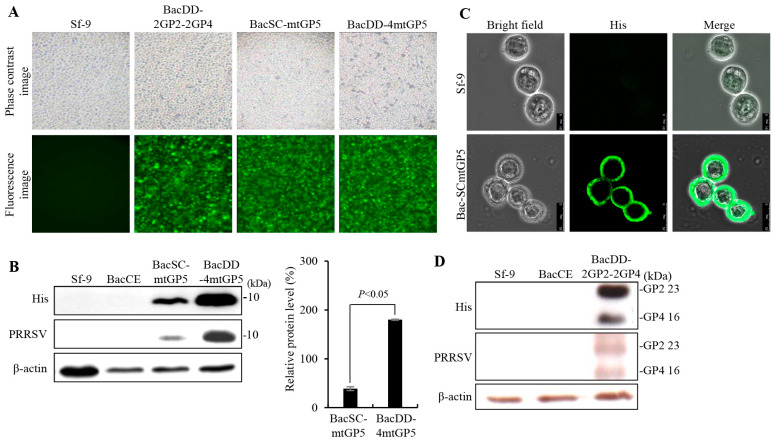
Construction of genetic recombinant baculoviruses BacSC-mtGP5, BacDD-2GP2-2GP4, and BacDD-4mtGP5. (**A**) Images of BacSC-mtGP5, BacDD-2GP2-2GP4, and BacDD-4mtGP5 baculovirus-infected SF-9 cells at 3 days post infection. Phase contrast images (upper panel) and fluorescence images (lower panel). All images were magnified at 200×. (**B**) Sf-9 insect cells were infected with the recombinant baculoviruses BacSC-mtGP5 and BacDD-4mtGP5, respectively, with an MOI of 20, harvested 3 days post infection, and subjected to Western blot using anti-His monoclonal antibody and PRRSV polyclonal antibodies, respectively. The ectodomain of His tagged mtGP5-gp64TM-CTD (10 kDa) fusion protein was detected. No proteins were detectable in the negative controls (BacCE and Sf-9 cell alone). The expected size of the His and baculovirus gp64TM-CTD fusion protein is approximately 5 KDa. Signals in Western blots were quantified using Image J software and shown in the right panel. (**C**) Anchoring of the ectodomain of His-tagged mtGP5-gp64TM-CTD fusion protein on the plasma membrane of Sf-9 cells as revealed by confocal microscopy. (**D**) Sf-9 cells were infected with the recombinant baculovirus BacDD-2GP2-2GP4 at an MOI of 20, harvested 3 days post infection, and then subjected to Western blot using anti-His monoclonal antibody and PRRSV polyclonal antibodies, respectively. BacCE and Sf-9 cells alone were used as negative controls. The expected sizes of His6-tagged GP2-gp64TM-CTD and His6-tagged GP4-gp64TM-CTD fusion proteins were approximately 23 kDa and 16 kDa, respectively. The expected sizes of the expressed fusion proteins are indicated on the right-hand side of each panel.

As shown in Figure 2A, three days after transfection, fluorescence signals were observed in BacSC-mtGP5, BacDD-2GP2-2GP4, and BacDD-4mtGP5 baculoviruses-infected Sf-9 cells. The EGFP was expressed in all transfected-Sf-9 insect cells and except un-transfected Sf-9 cells as expected. In order to confirm that the target protein is successfully expressed in insect cells, the cells were infected with the recombinant baculoviruses (an MOI of 20) BacSC-mtGP5, BacDD-4mtGP5, BacDD-2GP2-2GP4, BacDD-4GP5, BacSC-mtGP5, and BacCE (empty vector), respectively. Three days post infection, the recombinant baculovirus-transfected Sf-9 cells and Sf-9 alone cells (negative control) were collected for Western blotting. The results revealed that expression of the His-tagged mtGP5-gp64TM-CTD fusion protein of PRRSV with the expected size of 10 kDa could be detected using PRRSV polyclonal antibodies and His monoclonal antibodies (Figure 2B). The higher expression levels were observed for the His-tagged mtGP5-gp64TM-CTD fusion protein by the pBacDD-4GP5 construct with four expression cassettes (Figure 2B). The Western blot analysis showed that the expression level of the His-tagged mtGP5-gp64TM-CTD fusion protein by the pBacDD-4GP5 vector is about 3–4 times higher than that of the BacSC-mtGP5. To examine whether the mtGP5 mutant of PRRSV fused with the baculovirus.

The gp64 signal sequence and gp64TM-CTD were successfully displayed on insect cell membranes, the mtGP5 mutant protein was visualized by confocal microscopy using anti-His antibodies. The recombinant baculovirus BacSC-mtGP5 displaying the His6-tagged mtGP5-gp64TM-CTD fusion protein on the plasma membrane of Sf-9 cells were observed under confocal microscopy (Figure 2C). No fluorescence signals were detected in Sf-9 cells (negative control). In this work, co-expression levels of the His6-tagged GP2-gp64TM-CTD and His6-tagged GP4-gp64TM-CTD fusion proteins of PRRSV with the expected size of 23 kDa and 16 kDa, respectively, were detected using a PRRSV polyclonal antibody and a His monoclonal antibody, respectively (Figure 2D). A comparison of expression levels of these fusion proteins using recombinant baculovirus BacDD-2GP2-2GP4 revealed that the expression levels of His6-tagged GP2-gp64TM-CTD were slightly higher than that of His6-tagged GP4-gp64TM-CTD fusion protein. No proteins were detectable in the mock groups (Bac CE and Sf-9).

### 3.3. Optimal Conditions for Expressing the Truncated Viral Proteins of PRRSV

**Figure 3:**
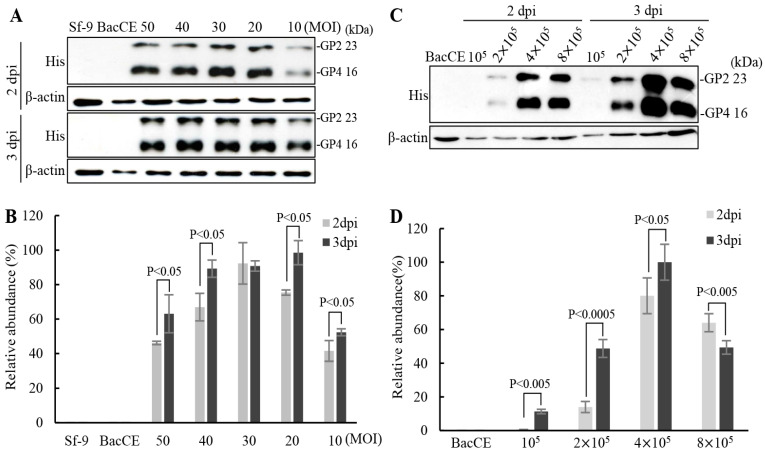
Optimum conditions for production of the truncated proteins of PRRSV. Effects of different infection times, MOI, and cell numbers for expressing the ectodomain of His-tagged GP2-gp64TM-CTD and His-tagged GP4-gp64TM-CTD fusion proteins of PRRSV were tested. (**A**,**C**) Sf-9 cells were infected with the genetic recombinant baculovirus BacDD-2GP2-2GP4 at different MOIs, infection times, and cell numbers. The Sf-9 cell alone and BacCE were used as negative controls. The expressed fusion proteins were probed using anti-His monoclonal antibodies. Signals in all Western blots were quantified using Image J software. β-actin was used as an internal control for normalization. Relative abundance (%) are shown. (**B**,**D**) The results were calculated from the data shown in panels A and C. All data shown represent the mean± SE calculated from three independent experiments.

**Figure 4:**
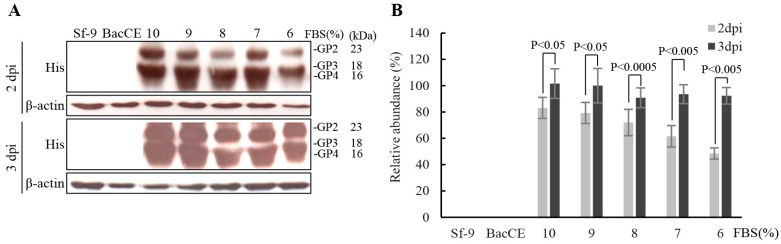
Effects of different concentrations of FBS on co-expression of the truncated fusion proteins of PRRSV. (**A**) Sf-9 cells were infected with genetic recombinant baculovirus BacD4D-GP2-GP3-2GP4 at an MOI of 20 supplemented with different FBS concentrations. The Sf-9 cell alone and BacCE were used as negative controls. The expressed proteins were probed using anti-His monoclonal antibodies. Signals in all Western blots were quantified using Image J software. β-actin was used as an internal control for normalization. Relative abundance (%) are shown. (**B**) The results were calculated from the data shown in the left panel. All data shown represent the mean± SE calculated from three independent experiments.

To increase the levels of expression of the PRRSV proteins and to reduce the production cost of subunit vaccines, the optimum conditions for recombinant baculovirus transfection and cell growth were optimized. The conditions of an MOI of 20 (BacD4D-2GP2-2GP4) and 3 day post infection showed higher expression levels of the ectodomain of His-tagged GP2-gp64TM-CTD and His-tagged GP4-gp64TM-CTD fusion proteins (Figure 3A,B). Furthermore, the expression of these fusion proteins of PRRSV in different cell numbers infected with genetic recombinant baculovirus BacD4D-2GP2-2GP4 with an MOI of 20 was examined. Our results revealed that higher levels of the truncated viral fusion proteins were observed in both BacD4D-2GP2-2GP4 and 4 × 10^5^ cell (Figure 3C,D). Furthermore, the expression of the ectodomain of His-tagged GP2-gp64TM-CTD, His-tagged GP3-gp64TM-CTD, and His-tagged GP4-gp64TM-CTD fusion proteins in different serum concentration culture conditions were assessed. Cells were infected with genetic recombinant baculovirus BacD4D-GP2-GP3-2GP4 with an MOI of 20 supplemented with different FBS concentrations (10%, 9%, 8%, 7%, and 6%). Our results reveal that higher expression levels of these truncated viral fusion proteins were observed in both 7% FBS and 3 day post infection (Figure 4A,B).

### 3.4. Expression of the Ectodomain of TrxA-His-GP2, TrxA-His-GP4, and TrxA-His-mtGP5 Fusion Proteins of PRRSV in E. coli BL21(DE3)

**Figure 5:**
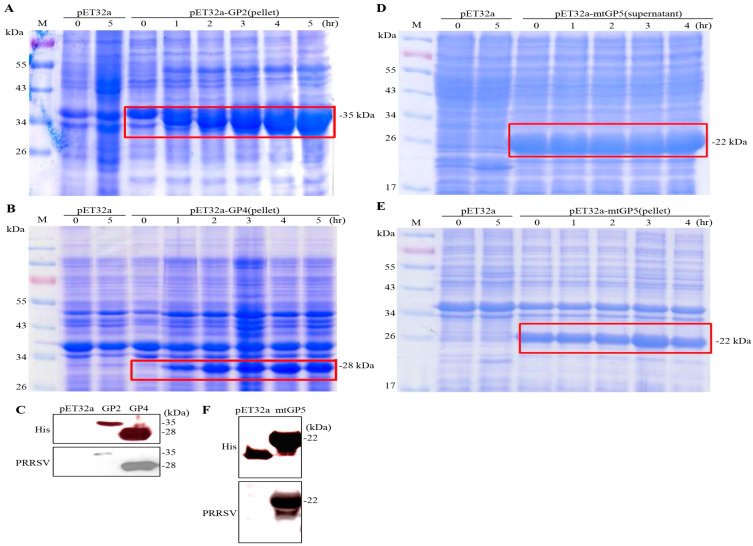
Coomassie blue-stained SDS-PAGE of E. coli BL21(DE3) expressing the PRRSV proteins. The PRRSV proteins were expressed as shown in panels A–E. (**A**) The ectodomain of TrxA-His-GP2, (**B**) TrxA-His-GP4, (**D**) TrxA-His-mtGP5 (supernatant), and (**E**) TrxA-His-mtGP5 (pellet). E.coli BL21 (DE3) containing the indicated constructs were induced for different time points with IPTG at a final concentration of 0.4 mM in culture medium. The pET32a (empty vector) was used as the negative control. The expressed proteins were marked by red frame. (**C**,**F**) The expressed TrxA-His-GP2, TrxA-His-GP4, and TrxA-His-mtGP5 fusion proteins were probed using anti-His monoclonal antibodies or PRRSV polyclonal antibodies, respectively. The expected size of the expressed fusion proteins is indicated on the right-hand side of each panel. The expected size of the TrxA-His fusion protein is about 17 KDa.

After confirming the gene sequences of pET32a-GP2, pET32a-GP4, and pET32a-GP5, the plasmids were transformed into *E. coli* BL21 (DE3), and a single colony was selected for amplification and culture. The expression of the ectodomain of TrxA-His-GP2 and TrxA-His-GP4 fusion proteins was induced by IPTG for 0–5 h. Our results showed that the expected sizes of the TrxA-His-GP2 fusion protein (insoluble protein) and the TrxA-His-GP4 (insoluble protein) were approximately 35 and 28 kDa (Figure 5A,B), respectively. The ectodomain of TrxA-His-GP2 and TrxA-His-GP4 fusion proteins were detected by Western blotting using His monoclonal antibodies and PRRSV polyclonal antibodies (Figure 5C). Our results showed that the expression levels of the ectodomain of TrxA-His-GP2 and TrxA-His-GP4 fusion proteins increased with the increase in induction times. As shown in Figure 5D,E, both the supernatant and the pellet had the ectodomain of TrxA-His-mtGP5 fusion protein with the expected size of 22 kDa. In addition, in order to confirm the antigenicity of the TrxA-His-mtGP5 fusion protein, Western blot assays were performed using anti-His monoclonal antibodies and PRRSV polyclonal antibodies. As shown in Figure 5F, the truncated TrxA-His-mtGP5 mutant protein was detected as expected.

### 3.5. Analysis of the Levels of Serum Antibodies and Neutralizing Antibodies in SPF Pigs Immunized with the Subunit Vaccine (BacD4D-2GP2-2GP4 and BacD4D-4mtGP5-Infected Cells)

**Figure 6:**
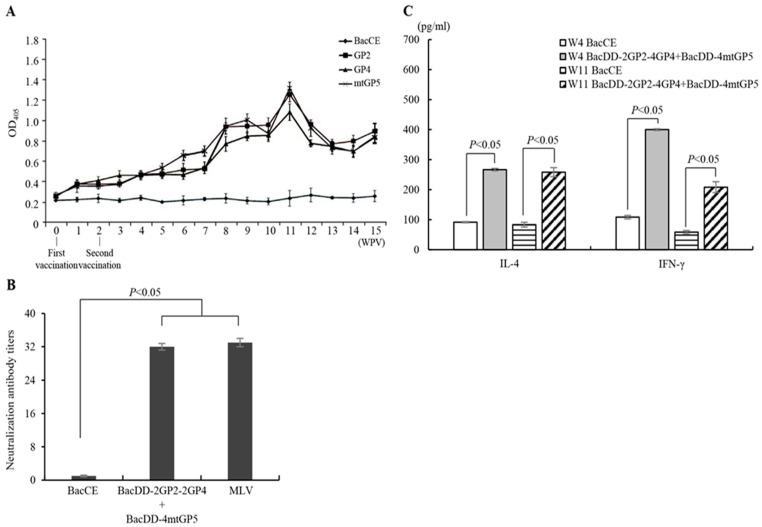
Detection of ELISA titers, SN titers, and the levels of IL-4 and IFN-γ in 5-week-old SPF pigs immunized with various immunogens. (**A**) Detection of anti-PRRSV proteins (GP2, GP4, and mtGP5) titers by the developed ELISA. The 2-month-old SPF pigs were immunized at the base of the ear by the intramuscular route with BacCE (negative control) and the developed subunit vaccine (prepared from BacD4D-2GP2-2GP4- and BacD4D-4mtGP5-infected cells, 5x10^7^ cells), respectively. Serum samples were collected every week after the first immunization to determine the GP2, GP4, and mtGP5 protein-specific ELISA antibodies. (**B**) SN titers in SPF pigs immunized with BaCE (negative control), subunit vaccine, and commercial MLV vaccine (positive control), respectively. Sera were from 11 weeks post vaccination. (**C**) Analysis of IL-4 and IFN-γ by ELISA was performed using IL-4 and IFN-γ ELISA kits. SPF pigs immunized with the developed subunit vaccine and BacCE (negative control), respectively. The levels of IL-4 and IFN-γ in the 4th and 11th weeks after the first and second immunizations were analyzed. The value of OD_405_ nm was measured with an ELISA reader. All data shown represent the mean ± SE calculated from three independent experiments.

In order to prepare an ELISA kit for detecting antibodies against viral proteins, the ectodomain of TrxA-His-GP2, TrxA-His-GP4, and TrxA-His-mtGP5 fusion proteins of PRRSV were expressed in E. coli BL21(DE3). After the purification of the expressed proteins, they were used as ELISA antigens. The optimal serum dilution factor is 128 folds. An ELISA-specific antibody analysis was performed under these conditions. In the group vaccinated with the developed subunit vaccine (prepared from BacD4D-2GP2-2GP4 and BacD4D-4mtGP5-infected cells), the antibody levels in the 16 weeks after the first and second immunization gradually increased and reached at a peak of about OD405 nm of 1–1.2, which was significantly different from the BacCE negative control group (Figure 6A). This result showed that the subunit vaccine indeed induces immune responses in SPF pigs. A diluted serum from pigs was mixed with virus in equal proportions, and after 1 h of neutralization reaction, it was added into a 96-well plate containing MARC-145 cells, and cultured for 3–5 days to observe cytopathic effect (CPE). Without neutralizing antibodies, PRRSV cannot be neutralized, the virus will infect MARC-145 cells, and CPE can be observed, which is non-neutralization. On the other hand, if there is a phenomenon of neutralizing the virus, no CPE will be generated, which is regarded as neutralization. Our results confirmed that the developed subunit vaccine can induce the higher levels of neutralizing antibodies, which in turn inhibit PRRSV infection. This result showed that the neutralizing antibody titers of the developed subunit vaccine and MLV vaccine were 32 folds, which was significantly higher than that of the BacCE negative group (Figure 6B).

### 3.6. Analysis of IL-4 and IFN-γ in Pigs Immunized with the BacD4D-2GP2-2GP4 and BacD4D-4GP5 Subunit Vaccines

A previous study showed that IFN-γ is an important factor in the vaccine-induced cellular immune response [48,49,50]. Therefore, IFN-γ was used as an indicator of cellular immunity in this study. It was confirmed by IFN-γ sandwich ELISA. Our results revealed that the developed subunit vaccine (BacD4D-2GP2-2GP4 and BacD4D-4GP5-infected cells) can stimulate immune cells to secrete IL-4 and IFN-γ. The results showed that SPF pigs immunized with the developed subunit vaccine could induce the production of IL-4 and IFN-γ. The amounts of IL-4 and IFN-γ could be measured in the serum at 270 pg/mL and 400 pg/mL at the fourth week after immunization, respectively (Figure 6C). There was a significant difference in BacCE in the negative group. The level of IFN-γ was gradually declined at the eleventh week after immunization while IL-4 level was not altered.

### 3.7. Preparation of Genetic Recombinant Baculoviruses BacDD-GP2-GP3-2GP4 and BacDD-2mtGP5-2M

**Figure 7:**
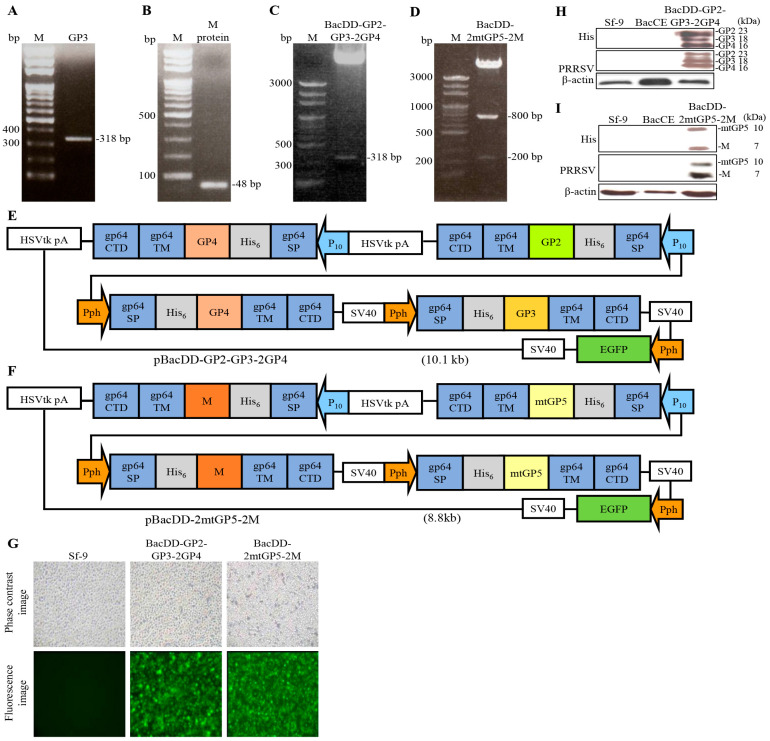
PCR amplification of the ectodomain sequences of GP3 and M genes of PRRSV and schematic illustration of baculovirus surface display vectors carrying multiple expression cassettes. (**A**,**B**) DNA electrophoresis analysis of PCR products of the ectodomain of GP3 and M genes of PRRSV. (**C**,**D**) The resultant pBacDD-GP2-GP3-2GP4 and pBacDD-2mtGP5-2M constructs digested with the respective restriction enzymes were analyzed by electrophoresis in a 1.2% agarose gel. (**E**,**F**) Schematic illustration of baculovirus surface display vectors pBacDD-GP2-GP3-2GP4 (**E**) and pBacDD-2mtGP5-2M (**F**), which carry multiple expression cassettes are shown. The baculovirus gp64TM-CTD and the signal peptide sequence of gp64 localized in the vectors are shown. The ectodomain of GP2, GP3, GP4, mtGP5, and M genes of PRRSV was inserted between the gp64 signal peptide and gp64TM-CTD of the surface display vectors under the control of the p10 or Pph promoter. (**G**) Sf-9 cells were transfected with the developed vectors to create recombinant baculoviruses BacDD-GP2-GP3-2GP4 and BacDD-2mtGP5-2M, respectively. Images of BacDD-GP2-GP3-2GP4 and BacDD-2mtGP5-2M recombinant baculovirus-infected Sf-9 cells at 3 days post infection are shown. All images were magnified at 200×. (**H**,**I**) To confirm that the target protein is successfully expressed in insect cells, the genetic recombinant baculoviruses BacDD-GP2-GP3-2GP4 and BacDD-2mtGP5-2M, and BacCE (empty vector) were used to infect insect cells at an MOI of 20 for three days, and the baculovirus-transfected Sf-9 cells and Sf-9 alone were collected for Western blot assays using PRRSV polyclonal antibodies and His monoclonal antibodies, respectively.

To further assess the co-expression efficiency of multiple viral genes, the pBacDD-GP2-GP3-2GP4 and pBacDD-2mtGP5-2M vectors were designed for creating genetic recombinant baculoviruses BacDD-GP2-GP3-2GP4 and BacDD-2mtGP5-2M, which simultaneously display the ectodomain of His-tagged GP2-gp64TM-CTD, His-tagged GP3-gp64TM-CTD, His-tagged GP4-gp64TM-CTD, His-tagged mtGP5-gp64TM-CTD, and His-tagged M-gp64TM-CTD fusion proteins on cell membrane of Sf-9 cells. To create the above constructs, the ectodomain coding sequences of GP3 and M genes of PRRSV were amplified by RT-PCR. PCR products were analyzed by electrophoresis in a 1.2% agarose gel. Our results showed that the target gene fragments were between 318 bp and 48 bp in size as expected (Figure 7A,B). The resultant pBacDD-GP2-GP3-2GP4 and pBacDD-2mtGP5-2M vectors were digested with the respective enzymes, respectively, to confirm the correctness of recombinant fragments. After an electrophoresis analysis in a 1.2% agarose gel, as shown in Figure 7C,D, the target gene fragments were 318 bp as well as 800 bp and 200 bp in size as expected. The schematic illustration of the resultant baculovirus surface display vectors with multiple expression cassettes are shown in Figure 7E,F. As shown in Figure 7G, three days after transfection, fluorescence signals were observed in BacDD-GP2-GP3-2GP4 and BacDD-2mtGP5-2M baculoviruses-infected Sf-9 cells. It could be seen that the EGFP was expressed in transfected-Sf-9 insect cells except for un-transfected Sf-9 cells. In order to confirm that the target protein is successfully expressed in insect cells, the genetic recombinant baculoviruses BacDD-GP2-GP3-2GP4, BacDD-2mtGP5-2M, and BacCE (empty vector) were used to infect insect cells at an MOI of 20 for 3 days, and the genetic recombinant baculovirus-transfected Sf-9 cells and Sf-9 alone were collected for Western blot assays. The results revealed that co-expression of the ectodomain of His-tagged GP2-gp64TM-CTD, His-tagged GP3-gp64TM-CTD, His-tagged GP4-gp64TM-CTD, His-tagged mtGP5-gp64TM-CTD, and His-tagged M-gp64TM-CTD fusion proteins with the expected size of 23, 18, 16, 10, and 7 kDa, respectively, could be detected using PRRSV polyclonal antibodies and His monoclonal antibodies, respectively (Figure 7H,I). Taken together, these results revealed that the developed baculovirus surface display vectors can simultaneously co-express two or three viral proteins.

## 4. Discussion

Within a few years of its emergence in the late 1980s, the PRRSV had spread globally to become the foremost infectious disease concern for the pork industry. Since 1994, modified live-attenuated vaccines against PRRSV have been widely used, but have failed to provide complete protection against emerging and heterologous field strains of the virus. Moreover, like many other MLVs, PRRSV-MLVs have safety concerns, including vertical and horizontal transmission of the vaccine virus and several documented incidences of reversion to virulence. Previous studies have pointed out that when the live virus vaccine virus strain remains in the animal, it undergoes homologous recombination with the field strain virus, which will cause the vaccine strain to restore the original pathogenicity of the virus [53]. Attenuated vaccines are still virulent and may infect weak piglets or pregnant sows and transmit the virus through body fluids and semen. Thus, the development of efficacious inactivated vaccines is warranted for the control and eradication of the PRRSV. Since the early 1990s, attempts have been made to develop inactivated PRRSV vaccines, however, most of the candidates have failed to elicit protective immunity even against homologous virus challenge. Recent reports relating to both inactivated and subunit candidate PRRSV vaccines have shown promise. However, these need to be pursued further to improve their heterologous efficacy and cost-effectiveness before considering commercialization. In this study, we offer PRRSV subunit vaccines and provide satisfactory immunogenicity studies in the pig model.

The baculovirus surface display vectors, including the baculovirus gp64 SP, gp64 TM-CTD, and multiple expression cassettes, have been constructed. Transcription is initiated by the pPH promoter or p10 promoter, and the target proteins are expected to form a fusion complex with the baculovirus gp64 TM-CTD. The gp64 TM-CTD can help the target protein to be presented on the Sf-9 cell membrane. In addition, the pPH promoter alone expresses EGFP, which can quickly observe the virus production under a fluorescent microscope, shorten the time for measuring the titer, and quickly determine whether the genetically recombined baculovirus is successfully obtained. The vector constructed in the present work contains five expression cassettes that can express multiple proteins and multiple sets of the same protein, respectively, thereby co-expressing different viral proteins and increasing the expression levels. The baculovirus surface display vectors have successfully been applied to prepare subunit vaccines [30,31,41,42,43], further supporting that the baculovirus surface display system has value for developing subunit vaccines. The GP2, GP4, and GP5 proteins of PRRSV are important antigens for inducing neutralizing antibodies [33,34,35]; therefore, we constructed two recombinant baculoviruses BacDD-2GP2-2GP4 and BacDD-4mtGP5. Importantly, the expression levels of mtGP5 by the pBacDD-4mtGP5 vector with four sets of expression cassettes were about 3–4 times higher than that of the BacSC-mtGP5 vector with one expression cassette, suggesting that the expression levels of the target protein could be elevated by this strategy. Furthermore, the developed pBacDD-2GP2-2GP4, pBacDD-GP2-GP3-2GP4, and pBacDD-2mtGP5-2M vectors simultaneously co-express the ectodomain of the PRRSV proteins, which confirms that multiple target proteins could be co-expressed by the developed baculovirus surface display system. SPF pigs immunized with the developed subunit vaccine showed that high levels of specific anti-PRRSV antibodies, neutralizing antibodies, IL-4, and IFN-γ could be elicited. In this work, the neutralizing antibody titers induced in SPF pigs by the developed subunit vaccine and MLV commercial vaccine were 32 folds, which was significantly higher than that of the negative group. Previous studies have suggested that the PRRSV GP5 protein mainly induces neutralizing antibodies against PRRSV [33,34,35,39], which is superior compared to other viral proteins [39]. The high levels of neutralizing antibody titers induction may be attributed to the substitution of the amino acid residue N34/51 with alanine (GP5N34A/N51A) located in GP5 to release the shielded epitopes on the GP5 protein of PRRSV. Taken together, our findings revealed that the developed baculovirus surface display system is a useful platform for the co-display of multiple proteins on the cell membrane of Sf-9 cells for preparing subunit vaccines against PRRSV.

A previous study suggested that the efficacy of using live cells for the preparation of a vaccine is better than that of the use of all cells (including dead cells). This may be related to the release of protease decomposed proteins after cell rupture [42]. Thus, the optimum conditions for co-expression of viral proteins were also assessed. Our results revealed that two days post infection under a MOI of 30 show higher expression levels of viral proteins. Since cells were rapidly infected with the high MOI of virus, live cells were harvested as a subunit vaccine before cells burst to death. The optimum conditions for co-expression of viral proteins by different genetic recombinant baculoviruses may show subtle differences and warrant modifications in the future.

There are two main mechanisms to induce an immune response after vaccination. One is that antigens are taken up by antigen-presenting cells (APCs) after immunization, and antigen fragments are presented to T cells by major histocompatibility complexes (MHCs). CD8^+^ T cells are mainly killer T cells, which can recognize the first class of MHC molecules and induce cytotoxicity and cytokine production [49]. CD4^+^ T cells are dominated by TH cells and can recognize the second class of MHC molecules. TH1 cells induce phagocytosis of cytokine-activated macrophages, and TH2 cells activate B cells to produce antibodies. Another is the direct binding of antigens to B cells to produce specific antibodies [54]. Previous reports have pointed out that IFN-γ is an indicator of vaccine-induced cellular immune response. CD4^+^ T cells secrete IFN-γ to activate CD8^+^ T cells and promote cytotoxicity, while activated CD8^+^ T cells continue to secrete IFN-γ, regulating cellular immunity [48,49,50]. A promising vaccine candidate must be able to induce both humoral and cellular immune responses. Our findings revealed that the PRRSV subunit vaccine prepared from BacDD-2GP2-2GP4 and BacDD-4GP5-infected cells could induce high levels of neutralizing antibody titers against PRRSV as well as the high levels of IL-4 and IFN-γ cytokines. The antibody titers of the developed subunit vaccine can last for more than 15 weeks. Previous studies have confirmed that neutralizing antibody titers up to 8 times can effectively inhibit viremia, and neutralizing antibody titers up to 16 times can avoid placental vertical infection of PRRSV [55]. The developed subunit vaccine can induce high levels of neutralizing antibody titers and IL-4 and IFN-γ cytokines in SPF pigs after immunization, suggesting that the subunit vaccine can induce both humoral and cellular immune responses against PRRSV infection. In summary, our developed polycistronic baculovirus surface display system offers an important platform to simultaneously co-express/display multiple viral proteins on cell membranes and to increase expression levels of PRRSV proteins for economical and effective subunit vaccine production.

## Data Availability

Not applicable.

## Supplementary Materials

The following supporting information can be downloaded at: https://www.mdpi.com/article/10.3390/vaccines11111666/s1, Supplementary Table S1. PRRSV primers used in this study.
