# Supplementary material for: Development of Polycistronic Baculovirus Surface Display Vectors to Simultaneously Express Viral Proteins of Porcine Reproductive and Respiratory Syndrome and Analysis of Their Immunogenicity in Swine
Source: Vaccines (Basel). 2023 Oct 31;11(11):1666. doi: 10.3390/vaccines11111666 (PMC10674950; doi:10.3390/vaccines11111666)
Supplement: Supplementary file 1 [file vaccines-11-01666-s001.zip › vaccines-2668280-supplementary.pdf]

Supplementary Table S1. PRRSV primers used in this study

Gene	Sequence (5'-3')*	Expected size (bp)
GP2	F: GCACGTCGACTCACCATCGCCGGTT(SalI)	444
	R: ACGGATCCACTCAAAGTACTATTATACAC(Bam HI)	
GP2	F: ACGCTAGCTCACCATCGCCGGTTGGCTG(Nhe I)	444
	R: ACATCGATACTCAAAGTACTATTATACAC(Cla I)	
GP3	F: GCACGTCGACTGCCTCACCCGGCAAGC(SalI)	318
	R: ACGGATCCTGAAATGTTGTCATGGCG(BamHI)	
GP3	F: ACCTCGAGTGCCTCACCCGGCAAGC(XhoI)	318
	R: CGAATTCTGAAATGTTGTCATGGCG (EcoRI)	
GP4	F: AACGCATGCCCATGTTTCAGTTCGAG(SphI)	267
	R: AACACCGGT TCCCTTTTCGCTCATCTC(AgeI)	
GP4	F: ACCTCGAGCCATGTTTCAGTTCGAG(XhoI)	267
	R: CGAATTC TCCCTTTTCGCTCATCTC(EcoRI)	
GP5	F:AACCTCGAGCCAAACACCAGCTCTTACTCCCAG(Xho I)	111
	R:AAATCTAGAACGCCCCAATGAAATTTGTC(Xba I)	
GP5	F:CCAACAACGCGGCCGCCCAAACACCAGCTCTTACTCCCAG(Not I)	111
	R: GCACGTCGACACGCCCCAATGAAATTTGTC(SalI)	
GP5	F: AACGCATGCCCAAACACCAGCTCTTACTCCCAG(SphI)	111
	R:AAAACCGGTACGCCCCAATGAAATTTGTC(Age I)	
GP5	F: AACAACCATATGCCAAACACCAGCTCTTACTCCCAG(Nde I)	111
	R:ACGCTAGCACGCCCCAATGAAATTTGTC(Nhe I)	
M	F:ACCTCGAGATGGGGTCGTCCCTAGACGAC(Xho I)	48
	R:CGAATTCCTGCGGAGCCGTGCT(EcoRI)	
M	F:AACACCATATGATGGGGTCGTCCCTAGACGCA(Nde I)	48
	R:ACGCTAGCCTGCGGAGCCGTGC(Nhe I)	

*Underlines indicate the location of restriction sizes.
